# Clinical deterioration after revisiting childhood sexual abuse during psychotherapy for restrictive anorexia nervosa: a case report

**DOI:** 10.3389/fped.2026.1958194

**Published:** 2026-10-14

**Authors:** Flavia Cirillo, Elena Bozzola, Giulia Spina, Mariangela Irrera, Alessandra Marchesi, Rosaria Marotta, Maria Rosaria Marchili

**Affiliations:** Pediatric Unit, Bambino Gesù Children’s Hospital, IRCCS, Rome, Italy

**Keywords:** adolescence, anorexia nervosa, childhood sexual abuse, developmental trauma, eating disorders, emotional regulation, trauma-informed care

## Abstract

**Introduction:**

Childhood traumatic experiences are increasingly recognized as important contributors to the development and maintenance of eating disorders. In adolescents with anorexia nervosa (AN), traumatic experiences may interact with restrictive symptoms through mechanisms involving emotional dysregulation, avoidance, and maladaptive control strategies. However, the optimal timing and approach for addressing traumatic experiences in severely underweight adolescents with AN remain unclear.

**Case presentation:**

We describe the case of a 16-year-old girl with restrictive AN who experienced a severe clinical relapse requiring hospitalization after a previously disclosed but unprocessed experience of childhood sexual abuse was revisited in greater detail during routine outpatient psychotherapy for AN. Although the psychotherapy was not trauma-focused, it aimed to reconstruct the patient's developmental history and identify the maintaining mechanisms of the eating disorder within a specialist multidisciplinary eating-disorder service. Revisiting the traumatic experience was followed by marked emotional distress and a severe exacerbation of restrictive symptoms.

**Conclusions:**

This case highlights the complex relationship between trauma and AN during adolescence and underscores the clinical importance of carefully assessing emotional regulation capacities and medical stability when traumatic experiences are revisited in severely vulnerable patients. A phase-based, trauma-informed multidisciplinary approach may be particularly important for adolescents whose restrictive symptoms continue to function as primary defensive and affect-regulatory mechanisms.

## Introduction

Anorexia nervosa (AN) is one of the most severe psychiatric disorders of adolescence and is associated with substantial medical morbidity, psychosocial impairment, and the highest mortality among psychiatric illnesses (1). Increasing evidence indicates that childhood traumatic experiences, particularly sexual abuse, emotional maltreatment, and neglect, are more common among individuals with eating disorders and are associated with greater symptom severity, psychiatric comorbidity, suicidality, and poorer treatment outcomes (2, 3).

Trauma may contribute to the development and maintenance of restrictive eating by promoting maladaptive strategies of emotional regulation. Food restriction, excessive control overweight, and rigid eating rituals may function as mechanisms of emotional numbing, experiential avoidance, and regulation of overwhelming internal states rather than reflecting concerns about body image alone (4). Developmental trauma models further suggest that early interpersonal trauma may disrupt emotional regulation, identity development, bodily integration, and interpersonal security, resulting in clinical presentations extending beyond classical post-traumatic stress disorder (PTSD) (5, 6). Although trauma-informed approaches are increasingly recommended in eating-disorder treatment, the optimal timing and modality of trauma exploration in medically fragile adolescents remain uncertain. Trauma-focused interventions have shown promising feasibility even in underweight patients, provided they are delivered within carefully structured, well-supervised therapeutic frameworks with continuous monitoring of emotional and nutritional stability (7–9).

The originality of the present case does not lie in the well-established association between childhood trauma and eating disorders. Rather, it concerns the temporal association between a severe relapse of restrictive AN and the detailed revisiting of a childhood sexual abuse experience that had previously been disclosed but never psychologically processed, during specialist psychotherapy for AN that was not formally trauma-focused. This observation raises clinically relevant questions regarding therapeutic pacing, emotional containment, and multidisciplinary communication when previously unprocessed traumatic material emerges in adolescents whose restrictive symptoms continue to serve a central affect-regulatory function.

This case report was prepared according to the CARE guidelines.

## Case presentation

The patient was a 16-year-old girl diagnosed with restrictive AN according to DSM-5-TR criteria. At hospital admission, her body mass index (BMI) was 16.3 kg/m^2^. Her physical parameters were: heart rate 60 bpm, blood pressure 104/62 mmHg, temperature 36 °C. She had no relevant medical, neurological, or neurodevelopmental history. From early childhood, she was described as emotionally sensitive, perfectionistic, compliant, and highly responsive to interpersonal expectations, with marked difficulty expressing emotional distress. The father suffered from an anxiety disorder. She was the second-born of three sisters. Her parents had separated 8 years prior and shared legal custody.

Restrictive eating began at 12 years of age, when her BMI was 22.6 kg/m^2^, with apparently health-oriented dietary changes that progressively evolved into severe caloric restriction, avoidance of multiple foods, calorie counting, excessive physical activity, intense fear of weight gain, cognitive rigidity, social withdrawal, and increasing preoccupation with body weight and shape.

At 13 years and 8 months, she required her first hospitalization after losing approximately 15 kg and developing sinus bradycardia (heart rate of 46 bpm) and malnutrition (BMI 16.9 kg/m^2^). A comprehensive medical evaluation excluded significant organic causes of the patient's progressive weight loss and malnutrition. Hematological, thyroid, gastrointestinal, inflammatory, and autoimmune investigations were unremarkable, including negative celiac screening and normal inflammatory markers and fecal calprotectin. Overall, the clinical and laboratory evaluation did not identify an alternative organic condition that could account for the progressive weight loss and nutritional deterioration. In the presence of persistent dietary restriction, intense fear of weight gain, excessive concern with body weight and shape, and associated restrictive behaviors, these findings supported the diagnosis of restrictive AN. She underwent multidisciplinary inpatient treatment including nutritional rehabilitation, psychiatric management, psychological support, and aripiprazole therapy, achieving partial weight restoration before discharge.

Following hospitalization, she entered a specialist public multidisciplinary eating-disorder service, where she remained in treatment from 2021 to 2024. Management included medical and nutritional monitoring, psychiatric follow-up, and individual psychotherapy specifically addressing AN. Although partial behavioral and nutritional stabilization was achieved, marked emotional vulnerability, fear of weight gain, cognitive rigidity, and excessive investment in body control persisted. She presented exaggerated emotional reactions even in apparently normal situations, a tendency to self-depreciation, difficulty in managing negative emotions and fear of external judgment, pessimism, negativism, discouragement and excessive guilt.

In early 2024, while still engaged in specialist outpatient treatment, previously disclosed but psychologically unprocessed childhood sexual abuse was explored in greater depth during psychotherapy. According to the patient's later retrospective account, discussing the abuse became progressively overwhelming and was followed by increasing emotional distress.

In July 2024, she experienced a severe relapse characterized by approximately 12 kg of weight loss over 6 months (BMI 16.26 kg/m^2^). She was prescribed escitalopram for the management of associated depressive and anxiety symptoms. She developed worsening mood, social withdrawal, and marked intensification of restrictive eating behaviors. The substantial weight loss and persistent restriction resulted in clinically significant nutritional and medical compromise, with inadequate oral intake and dehydration requiring close medical monitoring and nutritional stabilization. During medical monitoring, she presented with hypoglycemia (glucose 60 mg/dL). Persistent refusal of oral intake, mainly of carbohydrates, further complicated medical stabilization, necessitating hospitalization for intravenous fluids and electrolyte replacement to restore hydration and correct metabolic abnormalities. Temporary nasogastric feeding was subsequently introduced to ensure adequate nutritional intake and support progressive nutritional rehabilitation, with careful monitoring during refeeding because of the risk of metabolic complications. Secondary amenorrhea represented an additional manifestation of the prolonged state of energy deficiency. The main clinical events and their temporal sequence are summarized in Figure 1.

**Figure 1:**
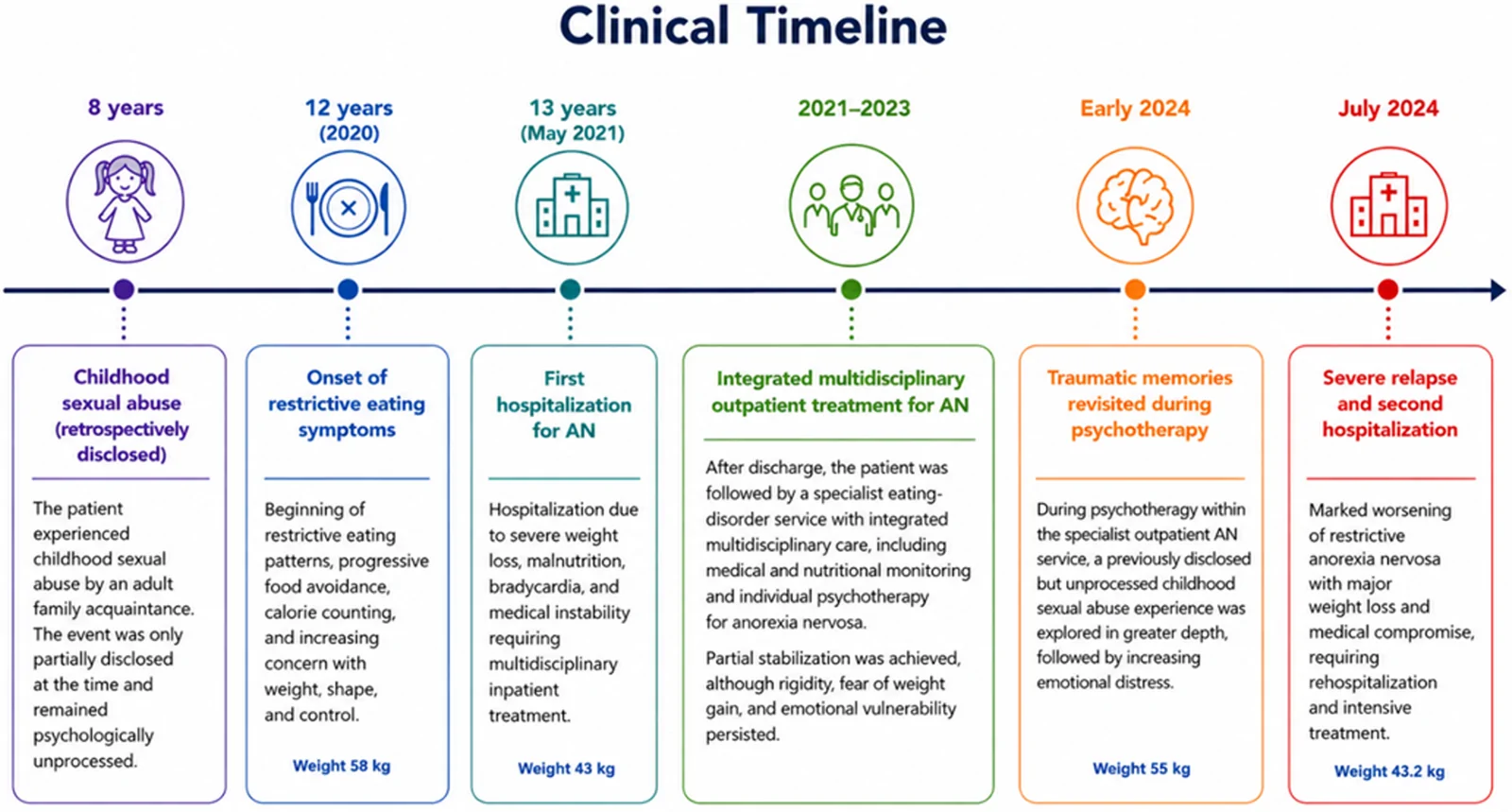
Clinical timeline of themainclinical and therapeutic events. This timeline represents a retrospective reconstruction based on patient interviews, parental collateral information, and available medical records.

## Diagnostic assessment

Laboratory and instrumental investigations performed during the second hospitalization excluded alternative organic causes of weight loss. Findings were consistent with malnutrition and included electrolyte abnormalities (potassium 3.2 mEq/L, phosphorus 2.9 mEq/L, sodium 132 mEq/L), mild thrombocytopenia (platelets 122,000/µL, reference range from 150,000 to 450,000/µL), elevated liver enzymes (alanine aminotransferase 90 U/L and aspartate aminotransferase 85 U/L, reference value <35 U/L), vitamin B1 deficiency (vitamin B1 24.4 ng/mL, normal value 32–95 ng/mL), and borderline vitamin D levels (vitamin D 29.3 ng/mL, reference range 30–100 ng/mL). During the second hospitalization, the patient retrospectively described the outpatient treatment received between 2021 and 2024. She reported that, while participating in specialist psychotherapy for AN within a multidisciplinary day-hospital program, she had been encouraged to explore emotionally significant life events and the developmental origins of her eating disorder. Within this therapeutic context, a childhood sexual abuse experience perpetrated by an adult family acquaintance when she was approximately 8 years old was revisited in greater depth. Although the abuse had been disclosed to her parents shortly after it occurred, it had remained only partially discussed and had never been psychologically processed. The patient described these sessions as progressively “too overwhelming,” particularly when invited to recall details of the abuse, reflect on its emotional meaning, and consider possible links with her eating disorder. According to her retrospective account, the exploration of the abuse was followed by increasing emotional distress, intrusive thoughts, shame, social withdrawal, and progressive intensification of restrictive eating behaviors, accompanied by a recurrent wish to “disappear.”

Following the patient's account of the traumatic experience, a comprehensive psychological and psychiatric assessment was conducted to further investigate comorbid psychopathology, trauma-related symptomatology, emotional regulation capacities, dissociative experiences, personality functioning, and perceived family functioning. The K-SADS interview identified depressive symptomatology associated with anxiety features (10). The Adolescent Dissociative Experiences Scale revealed no clinically significant dissociative symptoms across the domains of amnesia, depersonalization/derealization, absorption, or passive influence experiences (11). Similarly, the Personality Inventory for DSM-5 Brief Form did not identify clinically relevant maladaptive personality traits (12). Assessment of trauma-related symptoms yielded a more nuanced pattern. On the Impact of Event Scale–Revised, the patient demonstrated clinically significant levels of intrusion, avoidance, and hyperarousal, suggesting persistent trauma-related distress associated with the recalled abuse experiences (13). Conversely, scores on the Trauma Symptom Checklist for Children did not reach clinical thresholds across post-traumatic symptom domains, indicating the absence of a clinically significant post-traumatic symptom profile (14). The Difficulties in Emotion Regulation Scale revealed clinically significant emotional dysregulation, with marked difficulties in emotional acceptance, goal-directed behavior under emotional distress, emotional clarity, and overall emotion regulation (15). Borderline elevations were also observed in impulse control, emotional awareness, and access to adaptive emotion regulation strategies. Family functioning assessment using the Family Assessment Device revealed clinically significant difficulties in communication, behavioral control, and overall family functioning, whereas problem solving, family roles, affective responsiveness, and emotional involvement remained within non-clinical ranges (16). Overall, the psychometric findings suggested that the patient's difficulties were characterized less by dissociative phenomena or a fully structured post-traumatic symptom profile and more by trauma-related emotional dysregulation, persistent hyperarousal, and relational vulnerabilities. Clinical interviews, consistently with the psychometric findings, further suggested significant difficulties in emotional identification and symbolization. Emotional distress frequently appeared to be translated into concrete body-focused control strategies rather than verbally processed or mentalized. The patient demonstrated limited capacity to tolerate intense affective states and tended to manage psychological activation through withdrawal, restrictive rituals, and interpersonal avoidance. In this context, restrictive symptomatology was clinically conceptualized as a maladaptive regulatory strategy aimed at managing overwhelming emotional states and restoring a subjective sense of control.

Although no causal relationship can be established, the close temporal association between the emergence of previously unprocessed traumatic material and the subsequent clinical deterioration was considered clinically relevant in the overall case formulation. The chronology was reconstructed retrospectively by integrating repeated interviews with the patient, collateral information provided by her parents, and available medical documentation from previous hospitalizations and outpatient treatment. Verbatim session transcripts and detailed psychotherapy records were unavailable; therefore, it was not possible to reliably reconstruct or classify the specific psychotherapeutic techniques employed during individual sessions. Based on the patient's retrospective account, the therapeutic work involved exploration of emotionally significant life events, recall of aspects of the abuse, reflection on their emotional meaning, and consideration of possible links between these experiences and her eating-disorder symptoms. These elements should not be interpreted as evidence that a structured trauma-focused protocol was delivered. Statements presented in quotation marks reproduce the patient's own words.

## Therapeutic intervention

Before the second hospitalization, the patient had received continuous multidisciplinary treatment for AN, including psychiatric follow-up, nutritional monitoring, and individual psychotherapy that was not specifically designed as trauma-focused treatment.

During the second hospitalization, management prioritized medical stabilization and nutritional rehabilitation through close medical supervision, temporary enteral feeding, gradual reintroduction of oral nutrition, and psychopharmacological adjustment with sertraline while maintaining psychiatric monitoring.

Psychological support consisted of clinical interviews aimed at helping the patient identify, understand, and regulate overwhelming emotional states and at increasing her awareness of the possible association between emotional distress, the reactivation of traumatic memories, and the intensification of restrictive eating behaviors. Given her marked medical and emotional vulnerability, no structured trauma-focused intervention was undertaken, and direct trauma processing was postponed. Family members received psychoeducation and supportive interventions.

Given the patient's marked emotional and medical instability, direct trauma processing was postponed. Treatment instead focused on restoring physical safety, reducing emotional dysregulation, strengthening self-regulatory capacities, and preparing the patient for future trauma-informed psychotherapy once adequate medical and psychological stability had been achieved.

## Follow-up and outcomes

During hospitalization, the patient progressively improved adherence to nutritional treatment, laboratory abnormalities normalized, and emotional and behavioral stabilization gradually emerged. By discharge, she had resumed oral intake with substantially less resistance and no longer required enteral nutritional support. At discharge, her body weight was 46.6 kg, with a BMI of 17.0 kg/m^2^. Assessment of vital signs documented a heart rate of 67 bpm and blood pressure of 120/60 mmHg. Laboratory tests showed normalization of electrolytes and normal liver and kidney function. A multidisciplinary follow-up plan, including psychiatric care, nutritional monitoring, ongoing psychotherapy, and family-based interventions, was established. Given the clinical formulation developed during hospitalization, subsequent treatment adopted a trauma-informed, stabilization-oriented approach, prioritizing affect regulation, strengthening psychological resources, and careful monitoring of restrictive symptoms before any further direct exploration of traumatic experiences. At short-term follow-up, the patient maintained improved adherence to treatment and remained engaged in specialized child and adolescent mental health care. One year after discharge, her body weight was 49 kg, and her BMI was 18.0 kg/m^2^. Blood pressure was within the normal range (120/60 mmHg), and electrocardiography showed sinus rhythm with a heart rate of 85 bpm. Although restrictive psychopathology persisted, she showed greater willingness to recognize emotional distress and participate actively in treatment.

## Discussion

The principal clinical contribution of this case is the retrospectively reconstructed temporal association between the detailed exploration of a previously disclosed but psychologically unprocessed childhood sexual abuse experience during specialist psychotherapy for AN and a subsequent severe relapse requiring rehospitalization. Although a single retrospective case cannot establish causality, the observed sequence raises clinically relevant questions regarding the management of trauma in adolescents whose restrictive symptoms continue to represent their primary strategy for emotional regulation.

The association between childhood trauma and eating disorders is well established (2, 3). Likewise, restrictive eating has increasingly been conceptualized as a maladaptive strategy for affect regulation, emotional avoidance, and management of post-traumatic arousal rather than solely as a manifestation of body-image disturbance (4). In this patient, food restriction appeared to serve functions of emotional control, numbing, and interpersonal withdrawal. Her recurrent wish to “disappear” may therefore be understood within a broader developmental trauma framework, in which restrictive symptoms become protective responses to overwhelming affect rather than expressions of weight and shape concerns alone (5, 6).

The originality of this report does not lie in confirming the relationship between trauma and eating disorders, which is already well documented. Rather, it concerns the specific clinical circumstance in which previously unprocessed traumatic material emerged during psychotherapy that was designed to treat AN rather than to deliver structured trauma-focused treatment. Unlike protocols such as Imagery Rescripting, where trauma processing occurs within a carefully planned therapeutic framework supported by specific stabilization procedures, the trauma in this case emerged during routine psychotherapeutic exploration of developmental history and maintaining mechanisms of AN (7, 9). This distinction may be clinically relevant because it suggests that patients’ responses may depend not only on the traumatic content itself but also on the timing, therapeutic structure, and degree of multidisciplinary containment surrounding its exploration.

Several factors may have contributed to the observed deterioration. The traumatic experience had remained emotionally unprocessed for years despite its early disclosure. At the same time, the patient presented with marked alexithymia, chronic shame, and limited capacity to identify and regulate emotional states. Finally, restrictive eating continued to represent her principal defensive strategy. Within this context, the reactivation of traumatic memories may have exceeded her available regulatory capacities, resulting in increased psychological disorganization and further intensification of restrictive behaviors as an attempt to regain internal control (4, 6–8).

Based on this reconstruction, it may be hypothesized that, although treatment was formally multidisciplinary, the emotional activation associated with trauma exploration was not fully integrated into the ongoing medical and nutritional monitoring. This interpretation should be regarded as hypothesis-generating rather than evidence of deficiencies in the outpatient management. Other potential contributors to relapse should also be considered. Relapse in AN is a multifactorial process, with several demographic, clinical, psychological, and illness-related factors potentially contributing to its occurrence (17). Given the retrospective nature of this case, developmental factors related to adolescence, as well as environmental or interpersonal stress or snot systematically captured in the available clinical documentation, cannot be excluded as potential contributors to the observed deterioration. Similarly, the available retrospective information does not allow the possible contribution of medication-related factors to be reliably determined. The re-emergence and exploration of traumatic material should therefore be considered one clinically relevant factor within a potentially multifactorial relapse process rather than an isolated or sufficient causal explanation.

Notably, the patient did not meet full diagnostic criteria for PTSD. Instead, her presentation was characterized by chronic shame, emotional withdrawal, interpersonal vulnerability, and rigid body-focused control, features consistent with developmental trauma models (5). This observation suggests that clinically significant destabilization following trauma reactivation may occur even in the absence of formal PTSD when restrictive symptoms remain the individual's principal adaptive strategy. This case also highlights the potential importance of multidisciplinary communication when trauma-related material emerges during treatment. Based on the retrospective reconstruction, it is possible that the emotional activation associated with the exploration of traumatic experiences was not fully incorporated into the shared medical and nutritional formulation. This observation should not be interpreted as criticism of the treating clinicians, nor does the present report permit conclusions regarding the quality of outpatient care. Rather, it suggests that explicitly sharing the emergence of previously unprocessed traumatic material among psychotherapists, physicians, dietitians, and psychiatrists may facilitate earlier recognition of subtle changes in eating behavior, emotional regulation, and nutritional status, allowing timely adaptation of the overall treatment plan.

Current reviews recommend integrating trauma-informed approaches into eating-disorder treatment (18). The present case extends this literature by illustrating that clinically significant destabilization may also occur when traumatic memories emerge during psychotherapy that is not formally trauma-focused. Accordingly, this report does not argue against discussing traumatic experiences during treatment for AN. Instead, it underscores the importance of careful pacing, continuous assessment of emotional and medical stability, and close multidisciplinary collaboration whenever previously unprocessed traumatic material begins to surface.

These conclusions should be interpreted considering the methodological limitations inherent to retrospective single-case reports. The psychotherapeutic process was reconstructed from the patient's account, parental information, and available clinical documentation rather than direct observation or session transcripts. Consequently, the findings should be considered hypothesis-generating rather than evidence of a causal relationship. Nevertheless, this case highlights three clinically relevant issues that deserve further prospective investigation: the potential vulnerability associated with the emergence of previously unprocessed trauma during psychotherapy for AN, the importance of carefully titrating trauma exploration in medically fragile adolescents, and the potential value of integrating trauma-related information within multidisciplinary clinical decision-making.

## Patient perspective

The patient retrospectively described the exploration of the traumatic experience as progressively “too overwhelming.” She reported increasing emotional distress, intrusive thoughts, shame, and a tendency to withdraw from interpersonal relationships, together with a growing need to restrict food intake and are current wish to “disappear.” From her perspective, restrictive eating became increasingly intertwined with the management of emotional distress during this period. During the subsequent hospitalization and stabilization process, she gradually became more able to recognize and communicate her emotional distress and to engage more actively in treatment. She reported feeling understood in relation to the distress associated with having revisited a particularly difficult experience and perceived this recognition of her emotional suffering as helpful during the recovery process.

## Data Availability

The raw data supporting the conclusions of this article will be made available by the authors, without undue reservation.
